# Majority rule can help solve difficult tasks even when confident members opt out to serve individual interests

**DOI:** 10.1038/s41598-023-42080-7

**Published:** 2023-09-08

**Authors:** Kiri Kuroda, Mayu Takahashi, Tatsuya Kameda

**Affiliations:** 1 Center for Adaptive Rationality, Max Planck Institute for Human Development, Lentzeallee 94, 14195 Berlin, Germany; 2 Department of Social Psychology, The University of Tokyo, 7-3-1 Hongo, Bunkyo-ku, Tokyo, 113-0033 Japan; 3 Japan Society for the Promotion of Science, 5-3-1 Kojimachi, Chiyoda-ku, Tokyo, 102-0083 Japan; 4 Center for Experimental Research in Social Sciences, Hokkaido University, N10W7, Kita-ku, Sapporo, Hokkaido 060-0810 Japan; 5 Brain Science Institute, Tamagawa University, 6-1-1 Tamagawagakuen, Machida, Tokyo, 194-8610 Japan

**Keywords:** Psychology, Human behaviour

## Abstract

When sharing a common goal, confident and competent members are often motivated to contribute to the group, boosting its decision performance. However, it is unclear whether this process remains effective when members can opt in or out of group decisions and prioritize individual interests. Our laboratory experiment (n = 63) and cognitive modeling showed that at the individual level, confidence, competence, and a preference for risk motivated participants’ opt-out decisions. We then analyzed the group-level accuracy of majority decisions by creating many virtual groups of 25 members resampled from the 63 participants in the experiment. Whereas the majority decisions by voters who preferred to participate in group decision making were inferior to individual decisions by loners who opted out in an easy task, this was reversed in a difficult task. Bootstrap-simulation analyses decomposed these outcomes into the effects of a decrease in group size and a decrease in voters’ accuracy accruing from the opt-in/out mechanism, demonstrating how these effects interacted with task difficulty. Our results suggest that the majority rule still works to tackle challenging problems even when individual interests are emphasized over collective performance, playing a functional as well as a democratic role in consensus decision making under uncertainty.

## Introduction

Human groups often tackle difficult problems by seeking consensus, through voting, jury trials, and personal meetings. Such consensus decision making usually improves collective performance when there is an objective solution to the problem, and thus the accuracy of group outcomes is definable^1,2^. Majority and plurality voting are the most representative consensus rules in human societies; with these approaches groups can attain consensus more efficiently than when using the unanimity rule, while also reducing the risk of dictatorship^3,4^. Voting-like consensus is also found in herd behavior of non-human gregarious animals, such as nest migration of ants^5,6^ and honeybees^7^ and predator detection of fish shoals^8^. These animals attain such collective movements by following an action rule called quorum response, under which individuals are more likely to engage in a specific behavior when they perceive some number of others have already committed to the behavior^9–11^.

Consensus decisions usually target the group’s overall performance and presume that all members will participate in the decision process. However, what if all members are not required to contribute but can choose to opt in to consensus building for a common goal? A recent review on collective decision making^12^ argued that individuals who are more confident about their competence may be more inclined to contribute their judgments to the group. In contrast, less confident members may refrain from opting in, yielding a voting group composed of just relatively high-performing members^12^ (see ref.^13^ for a similar case). In fact, this claim has been supported by previous studies: Group decisions made by voluntary judgments were more accurate than those made by forced judgments^14,15^; accurate forecasters answered more questions than inaccurate forecasters^16^ and commented more frequently^17,18^; and more confident and competent members expressed their opinions earlier in the group situation^19,20^. Taken together, these results suggest that (1) the chance to opt in/out can increase the proportion of experts or well-informed members in the voting group, thus improving the collective performance, and that (2) members’ confidence and competence are the key cognitive parameters determining whether they opt in/out.

Although these studies have provided insights into the opt-in/out mechanisms and their effects on collective performance, their findings are based on the implicit assumption that all the members share a group goal. In daily life, however, individual interests are sometimes emphasized over group performance. Depending on whether people prioritize individual interests or group performance, they may join a group to survive (e.g., work in a company, become part of a couple) or work on life issues alone (e.g., do freelance work, remain single). The option to prioritize individual interests over group performance is particularly critical in human group decision making. In contrast to a colony of eusocial insects where kin selection guarantees the benefit of each individual’s contribution to the group, human groups often must navigate a trade-off between individualistic goals and collective performance^4^. Yet to our knowledge, no study has directly tested whether allowing group members to opt in/out still improves group performance when they have the option of pursuing individual interests by working alone.

Three questions arise when individual interests are at stake and people are free to opt in to or out of consensus decision making (here we focus on majority decision making). The first question concerns group members’ micro-decisions, and the second and third questions concern groups’ macro-outcomes resulting from the group members’ micro-decisions.

First, who opts out of majority decision making? Whereas confident and competent members often contribute to the group’s decisions when there is a collective goal^17–19^, improving the outcome (cf. ref.^20^), when individual interests are highlighted, they may be less motivated to join majority decision making. We thus hypothesized that more confident and competent members opt out of majority decision making when individual interests are emphasized over group performance. Aside from confidence and competence, we also conjectured that more risk-averse members are more likely to opt in to majority decision making because people may expect the group to reduce the noise of individual judgments and be more accurate than individuals^11,21–26^.

Second, which will be more accurate, the majority decisions made by voluntary participation (i.e., by those who opt in) or the individual decisions of loners (i.e., those who opt out)? Third, will the majority decisions made by voluntary participation be more accurate than those made by mandatory participation, where all members must join majority decision making? If more confident and competent members are more likely to opt out, the remaining members will be less accurate, thus deteriorating the accuracy of majority decisions. Hereafter, we refer to this negative group-level effect resulting from people voluntarily opting in/out as a “decrease in voters’ accuracy”. However, this is not a simple issue because group performance also depends on group size^27–30^ and task difficulty^31^. Therefore, we examined the accuracy of majority decisions while considering the effects of a decrease in voters’ accuracy as well as group size (“decrease in group size” through opting out) and task difficulty.

To address these questions, we conducted a laboratory experiment in which participants were asked to choose whether to opt in to or out of majority decision making. Participants performed a perceptual task^32^ with monetary incentives, which enabled us to dissociate their confidence from competence using techniques of cognitive modeling; participants’ risk preferences were estimated from a separate gambling task^33^. We set the maximum group size to 25 because the effect of group size on increasing collective performance marginally diminishes when the group has approximately 25 members (e.g., ref.^29^).

## Results

### Tasks

**Figure 1:**
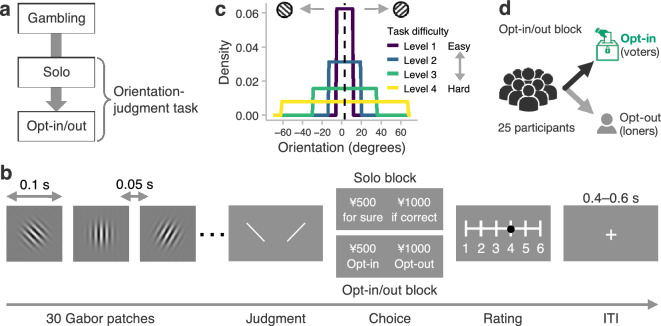
Experimental tasks. (**a**) Timeline of the experiment. Participants first performed the gambling task and then the orientation-judgment task composed of a solo and an opt-in/out block. (**b**) Trial flow of the orientation-judgment task. Participants observed 30 tilted Gabor patches and then judged whether the average orientation was tilted clockwise or counterclockwise. In the solo block, we asked participants to decide whether to bet on the perceptual judgment to obtain a reward or earn a sure but smaller gain, thus eliciting participants’ subjective accuracy. Participants also rated subjective confidence about the perceptual judgment on a 6-point scale. (**c**) The orientations of the stimuli in the orientation-judgment task. The orientations were sampled from a uniform distribution with mean, *m*, exactly + 3° or − 3°. This figure shows the case where *m* = + 3° (the vertical dashed line). The endpoints of the uniform distributions were *m* ± *v* (*v* = 8°, 16°, 32°, or 64°). The larger *v*, the harder the task (Supplementary Fig. S2). (**d**) Opt-in/out block. Participants performed the perceptual task and then chose to opt in or opt out. The majority decision on the average orientation was made among voters who opted in, out of the 25-person group. The voters’ rewards were determined by whether the majority decision was correct or wrong. For the loners who preferred to opt out, rewards depended on whether their own perceptual judgments were correct or wrong. *ITI* intertrial interval.

Sixty-three participants first performed the gambling task and then proceeded to the main perceptual task (orientation-judgment task) composed of a solo and an opt-in/out block (Fig. 1a). Participants’ risk preferences were estimated from the gambling task (see “Methods” section, Supplementary Fig. S1a, and Supplementary Table S1 for details of the task). We separately measured participants’ competence and confidence in the orientation-judgment task as explained below.

The orientation-judgment task was developed originally in a study on modeling human confidence^32^. Participants observed a series of 30 tilted Gabor patches and judged whether the average orientation of the stimuli was clockwise or counterclockwise relative to the vertical (“30 Gabor patches” and “Judgment” in Fig. 1b). To assess individual competence in the task and investigate how task difficulty affects collective performance, we manipulated the difficulty by generating the 30 orientations from uniform distributions with the same mean (+ 3° or − 3°) but different variances across trials (8°, 16°, 32°, or 64°; Fig. 1c shows the case where the mean was + 3°). Hereafter each variance is referred to by level (1–4); the higher the level (i.e., the greater the variance), the greater the difficulty (Supplementary Fig. S2).

The orientation-judgment task followed after participants judged the average orientation. In the solo block, participants decided whether to bet on their perceptual judgments for a larger reward or to obtain a fixed but smaller reward (“Choice” in Fig. 1b). Note that greater subjective accuracy about the perceptual judgment should make participants more willing to bet; that is, this procedure elicited participants’ confidence behaviorally (see the next section “Estimating cognitive parameters” for the definition of confidence in this paper). At the end of the trial, participants also reported their subjective confidence about their perceptual judgments on a 6-point scale (“Rating” in Fig. 1b).

In the opt-in/out block, each of the 63 participants decided whether to opt in (i.e., contribute their judgments) to majority decision making on the average orientation (“Choice” in Fig. 1b). Before the main experiment, we had run a separate experiment in which 24 other participants worked on the same perceptual task and decided whether to opt in or out of the majority decision making among them (see “Methods” section for details). Each of the 63 participants in the main experiment was grouped with these 24 previous participants (Fig. 1d). Participants in the main experiment were explicitly told that they would be grouped with 24 previous participants but did not know how many of them had decided to become voters in each trial. For the purpose of compensation to each participant in the main experiment (see “Grouping and payment procedure” in “Methods” section for details), the majority decision was made among the members of the 25-person group (i.e., the group comprising a participant plus the 24 previous participants) who chose to opt in for the trial (the “voters”). If the majority decision was correct, each voter in the main experiment earned 500 JPY (approximately 3.6 USD), otherwise, nothing. On the other hand, for the “loners,” who opted out of majority decision making, rewards depended on whether their own perceptual judgments were correct or wrong. The loners earned a reward greater than or equal to the 500-JPY reward of the voters if the judgment was correct but nothing if it was wrong. The amount of the reward for loners was varied across trials (see “Methods” section for details).

### Estimating cognitive parameters

To analyze how each participant’s risk preference, competence, and confidence affected their opt-in/out choices, we first estimated these cognitive parameters by fitting decision models to participants’ behavior as follows (see “Methods” section and Supplementary methods for full details): *Risk preference*^34^ (ρ) was estimated from the gambling task using the standard models in experimental/behavioral economics and other behavioral sciences (Eqs. 1 and 2; Supplementary Fig. S1b). A larger ρ indicates that the participant is willing to take more risks (Supplementary Fig. S1c). *Competence* (γ) was estimated by fitting the stochastic updating model^32^ (Eq. 3; Supplementary Fig. S3a left) to the perceptual judgments. The larger γ, the more competent the participant (Supplementary Fig. S3c). *Confidence* about the perceptual judgments was defined as the degree of deviation from the perfect calibration between objective and subjective accuracy^35–37^. Hereafter, each participant’s overall confidence in the task (i.e., subjective accuracy minus objective accuracy; Eqs. 4 and 5) is referred to as *c*, which is positive if the participant was overconfident, negative if underconfident (see the right panel in Supplementary Fig. S3a for illustration). That is, the larger *c*, the more confident the participant (Supplementary Fig. S3d). No significant correlation was found between the estimates of *c* and γ (competence), Pearson’s *r* = − 0.08, *P* = 0.549.

### Confident participants opted out of majority decision making

**Figure 2:**
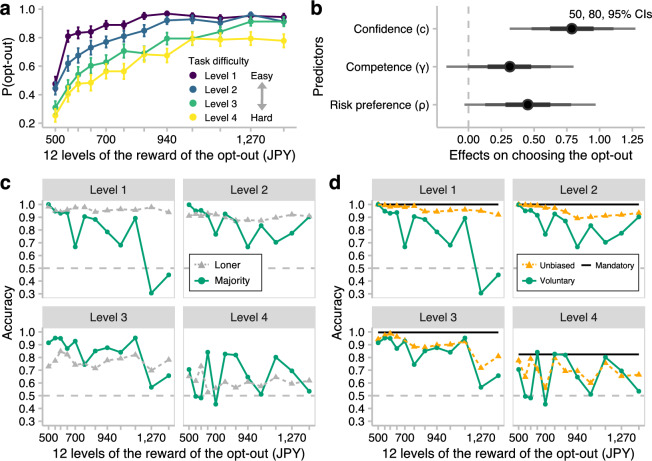
Results from the opt-in/out block. (**a**) Probability of opting out as a function of the task difficulty and reward for the opt-out choice. Participants opted out more frequently when the task was easier, and the reward was larger. The error bars indicate the standard errors of the means across participants. (**b**) Effects of the cognitive parameters on participants’ decision to opt out. The predictors affected the opt-out choice positively, but only for confidence did the 95% credible interval (CI) not contain zero. The points indicate the medians of the posterior distributions, and the error bars indicate the 50, 80, and 95% CIs. (**c**) Decision accuracy as a function of the task difficulty and reward for the opt-out choice: Majority decisions by voluntary participation versus loners’ individual decisions. Although the majority decisions by voluntary participation were worse than the loners’ individual decisions when the task was easier, the majority decisions began to outperform the individual decisions as the task got harder. The resampled data set in which no one voted was excluded from calculating the accuracy. (**d**) Accuracy of majority decisions as a function of the task difficulty and reward for the opt-out choice: Voluntary participation versus mandatory participation versus unbiased majority. The unbiased majority decisions were more accurate than the voluntary participation when the task was easy, but the difference in accuracy almost diminished as the task became difficult. For the mandatory participation, the accuracy is collapsed across the rewards of the opt-out choice because the 25 participants were treated as if all had chosen to opt in regardless of the reward.

After the parameter estimation, we addressed our first question about who would opt out of majority decision making. To examine the effects of the cognitive parameters on participants’ opt-in/out choices, we performed a hierarchical Bayesian logistic regression (Eq. 6). The response variables were participants’ opt-in/out choices (opt in = 0, opt out = 1), and the predictor variables of interest were participants’ risk preference, competence, and confidence (see Supplementary results and Fig. S4 for the correlations among the estimates of the cognitive parameters). Because participants’ opt-in/out choices clearly depended on the task difficulty and reward of the opt-out choice (Fig. 2a), we included them as control variables in the regression.

Figure 2b shows that confidence had a positive effect on participants’ opt-in/out choices (β = 0.788, 95% credible interval [CI] [0.314, 1.273]), indicating that when behaving as a loner could possibly yield larger earnings, more confident participants were more likely to opt out. Although risk preference and competence also affected the opt-in/out choices positively (i.e., risk-taking or competent participants were likely to opt out of majority decision making), the 95% CIs contained zero (risk preference: β = 0.452, 95% CI [− 0.031, 0.969]; competence: β = 0.315, 95% CI [− 0.169, 0.801]). For the control variables, see Supplementary results and Fig. S5.

These results support our prediction that confident people opt out of group decision making when individual rewards are involved. Importantly, this contrasts with previous findings that confident members are rather willing to contribute their judgments to a group when collective performance is emphasized^17–19^. Although not as strong as confidence, it is also noteworthy that risk preference affected the opt-in/out choices; that is, risk-averse participants tended to participate in majority decision making. This result suggests that participants may have expected the group to reduce the noise or variance of personal information^11,21–26^ and thus regarded opting in as a safer option.

Competent participants also tended to opt out of majority decision making (Fig. 2b). Their opt-outs could have degraded the average accuracy among the voters as compared to that of the original population composed of both voters and loners (cf. ref.^13^). Such a decrease in voters’ accuracy could have deteriorated the aggregated group performance, but was it true?

### Comparing decision accuracy

Given the above results, we focused on our second and third questions: (1) Which were more accurate, the majority decisions by voluntary participation or loners’ individual decisions? (2) Were the majority decisions by voluntary participation more accurate than those by mandatory participation? To address these questions, we performed the following bootstrap-simulation analyses by resampling participants’ behavior in the opt-in/out block, i.e., creating virtual groups.

#### Which were more accurate, the majority decisions by voluntary participation or loners’ individual decisions?

We calculated the decision accuracy of the majority decisions by voluntary participation and loners’ individual decisions by resampling participants’ behavior from the opt-in/out block as follows. First, for each combination of task difficulty (4 levels) and reward for opting out (12 levels), we randomly composed a 25-person group by sampling from the 63 participants. Second, as shown in Fig. 1d, the 25 participants were divided into voters or loners according to their choices in each trial. Finally, we checked whether the majority decision by the voters was correct. If only one participant voted, we took the participant’s decision as the majority decision; in case of a tie vote, the majority decision was determined randomly. We also calculated the loners’ accuracy (i.e., the proportion of correct loners). This bootstrap sampling was repeated 10,000 times for each combination of the task parameters to obtain more precise estimates of the accuracy.

Figure 2c shows the accuracy of the majority decisions by voluntary participation (the solid green lines) and loners’ individual decisions (the dashed gray lines) as a function of the task difficulty and reward for opting out. We make three observations. First, at Level 1 (i.e., the easiest level), the loners’ individual decisions outperformed the majority decisions if the reward for opting out was larger than that for opting in (500 JPY). A similar pattern can be observed at Level 2. Second, as the reward for opting out increased, the accuracy of majority decisions decreased, especially when the task was easier. Third, however, the differences in accuracy became less apparent as the task got harder (i.e., at Levels 3 and 4). In summary, the majority by voluntary participation was clearly inferior to the loners when the task was easy, whereas when the task was difficult, the majority decisions could be better than the loners’ individual decisions.

Two possible reasons could explain these results. The first account is based on the decrease in voters’ accuracy mentioned earlier (Fig. 2b): Confident and competent participants opted out of majority decision making when the task was easy, thus strongly degrading the voters’ average accuracy. The second explanation is based on group size (Fig. 2a): In the easy task, most participants preferred to opt out, and thus there were not enough voters (i.e., there was a decrease in group size) to reduce the noise of individual judgments. This point is addressed in the next section.

#### Were the majority decisions made by voluntary participation more accurate than those made by mandatory participation?

To address our third question, we compared the accuracy of majority decisions between voluntary and mandatory participation (see the section above for calculating the accuracy of voluntary participation). The accuracy of mandatory participation was computed using the same procedure as for voluntary participation, except that all 25 participants were treated as if they had been obliged to opt in to majority decision making.

Figure 2d shows the accuracy of the majority decisions by voluntary participation (the solid green lines) and mandatory participation (the solid black lines). As suggested by Condorcet’s jury theorem, the majority decisions by mandatory participation achieved a high accuracy (100%, 100%, 99.7%, and 82.4% for Levels 1–4, respectively). This result is not surprising because (1) the group consisted of 25 participants—that is, the group size was sufficiently large^29^, and (2) the group was composed of both voters and loners, and thus there was no decrease in voters’ accuracy, unlike in the majority decisions by voluntary participation. Nevertheless, as the task got harder, the difference in accuracy between voluntary and mandatory participation became smaller.

What factor contributed to the group-decision accuracy with voluntary participation? To address this, we further calculated the accuracy of another majority decision-making group that had the same group size as voluntary participation but was free from a decrease in voters’ accuracy. This accuracy was computed by (1) composing a 25-person group, (2) counting the number of voters among them, and (3) randomly assigning the role of voter to an equal number of participants irrespective of their real choices. Hereafter, this majority decision is referred to as an “unbiased majority decision”. By this definition, the difference in accuracy between the unbiased majority and the majority under mandatory participation reflects only the decrease in group size. On the other hand, the difference between the unbiased majority and the majority under voluntary participation indicates only the effects of a decrease in voters’ accuracy. That is, this analysis allowed us to dissociate the effect of a decrease in voters’ accuracy from the effect of a decrease in group size, in voluntary participation.

The accuracy of unbiased majority decisions is indicated by the orange dashed lines in Fig. 2d. One can see two patterns from the figure. First, when the task was easy (i.e., Levels 1 and 2), the unbiased majority decisions were not as inaccurate as those made by voluntary participation (the solid green lines) and were even comparable to those made by mandatory participation (the solid black lines). Second, however, the difference between the unbiased majority decisions and the voluntary majority decisions became smaller as the task got more difficult (i.e., in Levels 3 and 4). These results show that (1) the effect of a decrease in voters’ accuracy was much more noticeable than that of a decrease in group size when the task was easy, but (2) the effect of a decrease in voters’ accuracy almost vanished when the task became more difficult.

These results also provide insights into the mechanisms causing the majority decisions made by voluntary participation to be better or worse than the loners’ individual decisions depending on the task difficulty (Fig. 2c; recall that the previous section described how the collective performance could be explained in two ways). That is, (1) when the task was easy, the decrease in voters’ accuracy caused by voluntary participation clearly deteriorated the accuracy of majority decisions, but (2) as the task got more difficult, confident and competent participants’ opt-in choices removed the negative effect of the decrease in voters’ accuracy and simultaneously increased the group size via their participation (as shown in Fig. 2a), thus boosting the accuracy of the majority decisions.

## Discussion

Majority decisions are often more accurate than individual decisions. Previous studies have shown that the collective performance can be further improved by allowing members to decide whether to contribute their judgments to the group^12,17–19^. However, little is known about whether such opt-in/out mechanisms still improve the accuracy of majority decisions when individual interests are highlighted. We hypothesized that more confident and competent members are more likely to opt out of majority decision making (our first question) and examined how their micro-decisions lead to the groups’ macro-outcomes (our second and third questions).

First, as predicted, more confident participants were more likely to opt out of majority decision making; although not as strong as confidence, competence and a willingness to take risks also tended to affect their opt-out choice positively (Fig. 2b). These results indicate that members’ confidence has different effects on their opt-in/out choice depending on whether individual interests or group performance is emphasized (cf. refs.^17–19^). Note that whereas confidence and competence have often been behaviorally confounded in previous studies^19,20^, we estimated these dispositions separately from participants’ behavior using cognitive modeling techniques. This methodological advantage provides additional support for our results.

Second, the majority decisions made by voluntary participation were inferior to the loners’ individual decisions for the easy tasks, but the majority could be more accurate than the loners for the difficult tasks (Fig. 2c). Third, although the majority decisions made by voluntary participation were worse than those made by mandatory participation when the task was easy, the difference became less apparent as the task got harder (green vs. black lines in Fig. 2d). These results could be attributable to a decrease in group size and a decrease in voters’ accuracy. We thus addressed this point with bootstrap analyses, which enabled us to dissociate these two factors (green vs. orange and orange vs. black lines in Fig. 2d). We showed that the effects of a decrease in voters’ accuracy were remarkable for the easy tasks, but the effects of a decrease in voters’ accuracy as well as a decrease in group size were diminished for the difficult tasks because confident and competent participants opted in to majority decision making. These results suggest that majority decision making can still function as a measure to tackle difficult tasks even when some members are motivated to pursue individual interests.

Our results have important implications for research on collective intelligence and members’ confidence. Previous studies have shown that the Bayesian optimal aggregation of members’ judgments is to put larger weights on judgments of the members with greater confidence^38–40^ if confidence is expressed according to a common metric across members and if confidence indicates members’ competence accurately^41^. Indeed, when members have a common goal, this confidence-weighting rule may be achieved by the opt-in/out mechanisms that motivate confident members to contribute to the group^12,17,18^. However, when individual interests are highlighted as in our experiment, members’ voluntary participation could yield a decrease in voters’ accuracy particularly for the easy tasks. In this case, applying the confidence-weighting rule to the less confident members might result in more inaccurate group outcomes. Future research should examine the robustness of the confidence-weighting rule across various group settings.

There are several limitations in this study because of the experimental design. First, this study focused on one-shot situations only and allowed participants to choose to opt in or out in every trial, although people interact with others and groups repeatedly in daily life. Under such long-term relationships, it might be difficult to opt out of group decision making without any constraints. Further research is necessary to test the applicability of our results to broader situations. Second, related to the point above, we did not examine participants’ learning processes during the experiment. An interesting future direction will be to implement feedback on task performance and examine how people adjust their opt-in/out decisions through learning and whether they can further improve collective performance. Finally, we treated the opt-in/out decisions symmetrically; that is, participants were placed in a neutral position and then asked to make the opt-in/out decision. However, people have usually already opted in to or out of groups, and thus the real question would be whether to move from the current state to the other. In such cases, there is probably a status quo bias^42^ in deciding whether to opt in or out. This point could be examined by setting asymmetrical costs and benefits between opting in and opting out.

Before concluding this paper, we discuss the sample size and the significance of our findings. We acknowledge that the sample size in this study (i.e., 63 participants) may have been relatively small and that in the simulation analysis, resampling the same participants may have influenced the results. Increasing the sample size in future studies would enhance the reliability of the findings. On the other hand, it should be noted that the chosen sample size was sufficient to capture the distribution of participants’ cognitive parameters^32^ and investigate how individual differences in these dispositions affect the opt-in/out decisions. Also note that even if we had collected, for example, 250 individuals to form 10 real groups of 25 members, the additional information obtained would not necessarily outweigh the cost. We believe that a simulation analysis based on behavioral/cognitive parameters obtained from a real sample (as employed in this study) may be a more efficient way to examine the accuracy of group decision making than experimentally assessing actual group decisions with a large number of participants.

One could also argue that our results may seem obvious in the sense that the cognitive parameters (confidence, competence, and risk preference) should influence opt-in/out behavior. We agree that such general, qualitative relations may be obvious, and in fact, we predicted them. However, the quantitative relationships between these parameters and opt-in/out behavior and, most importantly, their aggregate impact on group-level outcomes cannot be known a priori, without empirical investigation coupled with cognitive modeling. Although we cannot eliminate the possibility that the variation between actual groups may be underestimated compared to virtual groups depending on the size of the individual data, we believe that systematic simulations of group-level behavior based on individual-level cognitive parameters is a useful research strategy to shed light on emergent collective phenomena.

In conclusion, we have experimentally demonstrated that the opportunity to opt in or out could motivate confident members to opt out of the group when individual interests are emphasized, and we have explained the group outcomes in terms of task difficulty, group size, and members’ accuracy. We believe that addressing individual interests and members’ opt-in/out decisions in future research is crucial for better understanding collective decision making in democratic societies that also respect individual independence.

## Methods

### Participants

Sixty-three university students with normal or corrected-to-normal vision participated in the experiment (32 men and 31 women; aged 19–28 years; right-handed). This sample size (*N* = 63) was determined by considering a previous study on modeling human confidence^32^ (*N* = 60 in total) with the same perceptual task (“30 Gabor patches”) as we used in this study. Thus, our sample size was comparable to that of the previous study and was sufficient to estimate the distribution of key cognitive parameters (confidence, competence) in the perceptual judgment. The experiment was approved by and carried out in accordance with the guidelines and regulations of the ethics committee of the Department of Social Psychology at the University of Tokyo (UTSP-19039). All participants gave written informed consent before the experiment.

### Stimuli

A set of PsychoPy^43^ scripts was used to control the experiment. Stimuli were presented on a 23.8-in monitor (1920 × 1080 resolution), 60 cm in front of the participant.

### Experimental procedure

#### Overview

Participants were individually invited to perform the gambling task and then the orientation-judgment task consisting of the solo and opt-in/out blocks (Fig. 1a). At the end of the experiment, participants received a 300-JPY show-up fee with an additional amount based on three randomly selected trials from a randomly selected task (see “Grouping and payment procedure” below). No information was given to participants during the tasks about their performance or the outcomes of their choices.

#### Gambling task

To measure participants’ risk preference, we used the task of ref.^33^ with a modified set of task parameters. Participants chose a sure or risky option 47 times. The sure option guaranteed 500 JPY, whereas the risky option yielded a larger reward with probability *p*_reward_, but nothing with probability 1 − *p*_reward_. The reward magnitude and probability of the risky option were indicated by a pie chart and varied across trials (Supplementary Fig. S1a and Table S1).

#### Orientation-judgment task

To quantify participants’ competence and confidence, we used the orientation-judgment task, which was devised by researchers modeling human confidence^32^ (“30 Gabor patches” and “Judgment” in Fig. 1b). Participants observed a series of 30 tilted Gabor patches, with each stimulus presented for 0.10 s with an interval of 0.05 s. Participants then judged whether the average orientation was tilted clockwise or counterclockwise relative to the vertical. The position of the alternatives was randomized across trials; participants selected the left or right alternative with the Q or P key, respectively, on a keyboard.

The orientations of the Gabor patches (Gaussian envelope *SD* = 0.63°; spatial frequency: 1.57 cycles/degree; contrast: 100%) were determined by uniform distributions with mean *m* and endpoints *m* ± *v* (Fig. 1c). We set two levels for *m* (+ 3° and − 3°), and four for *v* (8°, 16°, 32°, and 64°). A larger *v* indicates a higher difficulty (Fig. S2). The 30 orientations were pseudo-randomly sampled such that the mean was exactly + 3° or − 3° in each trial. After participants judged the average orientation, they proceeded to the orientation-judgment task’s solo and opt-in/out blocks.

##### Solo block

In the solo block (96 trials), participants chose a sure or risky option: The sure option promised 500 JPY regardless of the perceptual judgment, whereas the risky option brought a larger reward if the judgment was correct but nothing if it was wrong (“Choice” in Fig. 1b). The reward of the risky option was varied across trials (550, 590, 640, 700, 770, 850, 940, 1,040, 1,150, 1,270, 1,400, or 1,540 JPY) and was jittered every trial by adding an amount randomly sampled from {− 10, 0, 10}. The spatial configuration of the options was counterbalanced; the left (right) option corresponded to the Q (P) key.

After the choice, participants rated their subjective confidence about the perceptual judgment on a 6-point scale (1 = *not confident at all*, 6 = *very confident*; “Rating” in Fig. 1b). An initial point in the scale was randomized across trials, and participants moved a cursor on the scale to the left or right with the Q or P key. Participants then confirmed their rating with the space key, followed by an intertrial interval of 0.4–0.6 s with a fixation cross. Participants experienced each combination of the task parameters once (96 trials = 2 levels of mean orientation × 4 levels of difficulty × 12 levels of reward).

##### Opt-in/out block

In the opt-in/out block (96 trials), participants chose whether to opt in to majority decision making (“Choice” in Fig. 1b). For the purpose of instantaneous payment at the end of the experiment, each participant in the main experiment was later grouped with 24 previous participants in a separate experiment, who had already chosen to opt in or out (Fig. 1d; for background on using this grouping procedure, see “Grouping and payment procedure” below). The majority decision on the average orientation was made among voters who chose to opt in for the trial, of the 25-person group. Although participants were explicitly told that they would be matched with the 24 previous participants, no information was given to them about the number of voters in each trial, to avoid suggesting any hints about the collective performance. If the majority of the voters were correct, each voter in the main experiment earned 500 JPY, but otherwise the voters received nothing. In contrast, for the loners who chose to opt out, their rewards were determined by whether their own perceptual judgments were correct or wrong. The reward when opting out was varied across trials (500, 550, 590, 640, 700, 770, 850, 940, 1040, 1150, 1270, or 1400 JPY) and was jittered as in the solo block. The position of the options was counterbalanced across trials. At the end of the trial, regardless of whether participants had opted in or out, they were asked to rate how accurate the majority decision would be on the 6-point scale (“Rating” in Fig. 1b). Because these rating data were measured exploratorily, the results are not reported in this paper. As in the solo block, participants experienced each combination of the task parameters once.

#### Grouping and payment procedure

To implement the majority decision making in the opt-in/out block (Fig. 1d) and pay the participants immediately after the experiment, we had conducted a separate experiment in which a different sample of 24 university students also chose to opt in or opt out. The 63 participants in the main experiment were explicitly told that they would be grouped with the 24 previous participants, so this study involved no deception.

At the end of the experiment, participants received a 300-JPY show-up fee with an additional amount based on three randomly selected trials from a randomly selected task. If the opt-in/out block was chosen, the participant was grouped with the previous 24 participants. The data from the separate experiment were used only to enable the payment procedure and were not included in the analysis.

### Model fitting

Participants’ individual cognitive parameters were estimated using hierarchical Bayesian methods (see Supplementary methods for details). The median of the posterior samples was used as each participant’s individual estimate of the parameter.

#### Risk preference

Risk preference was estimated from participants’ choices in the gambling task (i.e., sure 500 JPY vs. a larger reward with probability *p*_reward_, but nothing with probability 1 − *p*_reward_). A participant was assumed to have a power utility function:

1 $$\begin{array}{c}u\left(x\right)={p}_{\mathrm{reward}}{r}^{\rho },\end{array}$$

where *u*(*x*) is the utility of option *x*, *p*_reward_ is the reward probability, *r* is the reward magnitude, and ρ is the participant’s risk preference. The value of ρ is less (greater) than 1 if the participant is risk averse (risk seeking). See Supplementary Table S2 for the model’s performance of the power utility function compared to the mean–variance utility function (Supplementary Eq. S1). The softmax (logistic) function of the utility of the options governs the participant’s probability of choosing the risky option:

2 $$\begin{array}{c}\begin{array}{c}P\left(\mathrm{risky}\right)=\frac{1}{1+\mathrm{exp}\left\{-\tau \left(u\left(\mathrm{risky}\right)-u\left(\mathrm{sure}\right)\right)\right\}},\end{array}\end{array}$$

where τ, the inverse temperature, controls the choice randomness. Because τ is just the scaling parameter in the participant’s choices and thus not the parameter of our interest, the results on τ are not reported in this paper.

#### Competence

Participants’ competence in the orientation-judgment task was estimated using the stochastic updating model, which was devised and validated by a study on human confidence^32^. This model assumes that participants updated the noisy estimate of the orientation after each stimulus presentation and made a perceptual judgment according to the sign of the final estimate (Supplementary Fig. S3a, left). The updating process is given by

3 $$\begin{array}{c}{\mu }_{i}={\mathrm{\lambda \mu }}_{i-1}+{\theta }_{i}+\varepsilon {\theta }_{i}{\xi }_{i},\end{array}$$

where μ_*i*_ is the estimate of the orientation after seeing up to the *i*th stimulus, λ > 0 is the weighting of past estimates relative to the current input, θ_*i*_ is the orientation of the *i*th stimulus in degrees (positive if clockwise, negative if counterclockwise), and ξ_*i*_ is noise from the standard normal distribution. Although we modified the original expression and notation following ref.^32^ to stabilize the parameter estimation and simplify interpretation of the parameters, the content of the model remains unchanged. The noise intensity is regulated by ε > 0, indicating that a smaller ε causes more accurate judgments. To interpret the parameter easily and index each participant’s competence, we reversed the sign of ε and refer to it as γ. The larger γ, the more competent the participant (Supplementary Fig. S3c). See ref.^32^ for the details of the procedure for estimating ε and λ.

#### Confidence

Confidence about the perceptual judgments was defined as the degree of deviation from the perfect calibration between objective and subjective accuracy^35–37^. For each participant and each trial, the objective accuracy (*p*) was numerically computed from the individual estimates of ε and λ by following ref.^32^. This *p* is equivalent to the “expected perceived probability of being correct” in ref.^32^.

We modeled the calibration between objective and subjective accuracy using the probability weighting function^44^ (the right panel in Supplementary Fig. S3a):

4 $$\begin{array}{c}w\left(p\right)=\frac{\mathrm{\alpha }{p}^{\beta }}{\mathrm{\alpha }{p}^{\beta }+{\left(1-p\right)}^{\beta }}=q,\end{array}$$

where α captures the elevation of the function (i.e., the greater α, the more confident the participant) and β controls the curvature of the function. The output of the function, *q*, was defined as the participant’s subjective accuracy in the trial (see Supplementary Table S3 for the model performance of Eq. 4 compared to five other probability weighting functions: Supplementary Eqs. S2–S6). Given this subjective accuracy, *q*, the participant was assumed to make risky decisions in the solo block [i.e., sure 500 JPY vs. a larger reward if correct (probability *q*) and nothing if wrong (probability 1 − *q*)] by comparing the utility of each option (Eqs. 1 and 2; Supplementary Fig. S1b).

To index the participants’ overall confidence in the task (i.e., the degree of deviation from the perfect calibration between objective and subjective accuracy), we integrated the area surrounded by *w*(*p*) and the 45° line (*q* = *p*, which indicates perfect calibration; see the right panel in Supplementary Fig. S3a for illustration):

5 $$\begin{array}{c}c={\int }_{0.5}^{1}\left(w\left(p\right)-p\right)dp.\end{array}$$

Note that the objective accuracy, *p*, ranges from 0.5 to 1 because *p* is conditional on the perceptual judgment, and thus *p* cannot be less than chance level, 0.5 (see ref.^32^ for details). Supplementary Fig. S3d shows each participant’s calibration curve between the objective and subjective accuracy.

### Statistical analysis

We performed a logistic regression (Eq. 6) and ordered logistic regression analyses using hierarchical Bayesian methods (see Supplementary methods for details). For the ordered logistic regression on confidence ratings, see Supplementary methods (Supplementary Eqs. S7 and S8) and Fig. S6. All the *P* values were two-sided, and the significance level was set at 5% for the null hypothesis tests.

To examine how participants’ cognitive parameters and the task parameters affected their opt-in/out choices, we ran a logistic regression, which predicts participant *i*’s probability of choosing to opt out at trial *t*:

6 $$\begin{array}{c}\mathrm{logit}\left({P\left(\mathrm{opting\ out}\right)}_{i,t}\right)={\beta }_{1,i}+{\beta }_{r,i}{Z}_{r,i,t}+{\beta }_{v,i}{Z}_{v,i,t}+{\beta }_{\rho }{Z}_{\rho ,i}+{\beta }_{\gamma }{Z}_{\gamma ,i}+{\beta }_{c}{Z}_{c,i}+{\beta }_{\lambda }{Z}_{\lambda ,i},\end{array}$$

where *r* is the reward for opting out, *v* is the variance of orientations (i.e., task difficulty), *Z*_(.)_ is the normalized value of each variable, β_1_ is the intercept, and the other βs are the coefficients for each *Z*.

### Posterior predictive checking

We performed posterior predictive checking to visually examine the fit of the cognitive and statistical models to participants’ behavior. See Supplementary methods and Figs. S7 and S8 for details.

### Supplementary Information

Supplementary Information.

## Data Availability

The data that support the findings of this study have been deposited in the Open Science Framework (https://osf.io/9ypv4/).

## Code availability

The codes used for the analysis have been deposited at Open Science Framework (https://osf.io/9ypv4/).
