# Supplementary material for: Majority rule can help solve difficult tasks even when confident members opt out to serve individual interests
Source: Sci Rep. 2023 Sep 8;13:14836. doi: 10.1038/s41598-023-42080-7 (PMC10491809; doi:10.1038/s41598-023-42080-7)
Supplement: Supplementary file 1 — Supplementary Information. [file 41598_2023_42080_MOESM1_ESM.pdf]

Supplementary Information for “Majority rule can help solve difficult tasks even when confident members opt out to serve individual interests”

Kiri Kuroda[1,3], Mayu Takahashi[2,3], and Tatsuya Kameda[2,4,5*]

1. Center for Adaptive Rationality, Max Planck Institute for Human Development, Lentzeallee 94, 14195 Berlin, Germany.
2. Department of Social Psychology, The University of Tokyo, 7-3-1 Hongo, Bunkyo-ku, Tokyo, 113-0033 Japan.
3. Japan Society for the Promotion of Science, 5-3-1 Kojimachi, Chiyoda-ku, Tokyo, 102-0083 Japan.
4. Center for Experimental Research in Social Sciences, Hokkaido University, N10W7, Kita-ku, Sapporo, Hokkaido, 060-0810 Japan.
5. Brain Science Institute, Tamagawa University, 6-1-1 Tamagawagakuen, Machida, Tokyo, 194-8610 Japan.

*Corresponding author: tkameda@l.u-tokyo.ac.jp

This PDF includes:

Supplementary Results

Supplementary Methods

Supplementary Figures S1–S8

Supplementary Tables S1–S4

Supplementary Results

Individual estimates of risk preference

For each participant, we estimated risk preference, ρ, from the gambling task (see Methods and Supplementary Fig. S1b). If ρ is less (greater) than 1, the participant is risk averse (risk seeking). The average of participants' ρ was 0.81 and significantly less than 1, indicating that participants were generally risk averse (Supplementary Fig. S1c; $M = 0.81$, $SD = 0.22$; one-sample t test: $t_{(62)} = -7.00$, $P < .001$, Cohen's $d = -0.88$, 95% confidence interval [CI] [−1.18, −0.59]). This risk-averse tendency is consistent with the results from a large-scale study on the methodology of risk elicitation[1].

No significant differences in accuracy in the orientation-judgment task between the blocks

We checked whether there were differences in the accuracy of the perceptual judgments between the solo and opt-in/out blocks (Supplementary Fig. S3b). The accuracy was calculated for each block, each level of variance (Fig. 1c), and each participant. We analyzed these values using a two-way (2 Blocks × 4 Levels of Variance) repeated-measures analysis of variance (ANOVA) as in ref.[2]. The ANOVA (with Greenhouse–Geisser correction) showed that the main effect of block was not significant, $F_{(1.00, 62.00)} = 0.67$, $P = .417$, $\eta_p^2 = 1.97\text{e}{-03}$, 95% CI [0.00, 0.02]; the interaction effect was also not significant, $F_{(2.50, 155.03)} = 0.52$, $P = .636$, $\eta_p^2 = 1.47\text{e}{-03}$, 95% CI [0.00, 0.01]. The main effect of the variance level was significant, $F_{(2.52, 156.04)} = 424.63$, $P < .001$, $\eta_p^2 = 0.29$, 95% CI [0.23, 0.35], indicating that the larger the variance, the more difficult the task (see also Supplementary Fig. S2). Since there were no significant differences between the blocks, we decided to use data from both blocks in fitting the stochastic updating model[2] (Eq. 3) to participants' perceptual judgments.

Participants' confidence in the orientation-judgment task

Supplementary Fig. S3d shows that most participants were underconfident, which is in line with previous studies on confidence using incentivized tasks[3–5]. Furthermore, as shown in Supplementary Table S4, our q modeled by Eq. 4 (i.e., subjective accuracy)[6] predicts participants' confidence ratings better than p (i.e., the objective accuracy), which was calculated from individual estimates of ε and λ. For 62 of 63 participants, q had a positive effect on their confidence ratings (Supplementary Fig. S6). These results provide additional support for our modeling of participants' subjective accuracy and confidence calibration.

No significant correlations among the estimates of cognitive parameters

Supplementary Fig. S4 shows the 10 Pearson correlations among participants' estimates of the cognitive parameters: ρ (risk preference; Eq. 1), λ (weighting of the past estimate relative to the current input; Eq. 3), γ (= minus ε, i.e., competence; Eq. 3), α (elevation of the probability weighting function; Eq. 4), and β (distortion of the probability weighting function; Eq. 4). After Holm–Bonferroni correction, we found no significant correlations among these estimates.

Effects of λ and the task parameters on participants' opt-in/out choices

In the logistic regression predicting participants' opt-in/out choices (Eq. 6), we entered the predictors: participants' ρ, γ, c, and λ, the reward for the opt-out choice, and the variance of orientations. For the coefficients for ρ, γ, and c, see Results and Fig. 2b in the main text.

The parameter λ, which is not a parameter of interest, had a positive effect on participants' opt-in/out choices on average, but the 95% credible interval contained zero ($\beta = 0.198$, 95% credible interval [−0.291, 0.694]). Supplementary Fig. S5 shows each participant's posterior distribution of the intercepts and the coefficients for the reward and variance. As seen in Fig. 2a, for most participants, the reward for opting out had a positive effect, and the variance (i.e.,

task difficulty) had a negative effect on their opt-in/out choices.

Supplementary Methods

Hierarchical Bayesian methods

We used Markov chain Monte Carlo (MCMC) and hierarchical Bayesian methods in the analysis. These methods allowed us to evaluate individual differences while they are bounded by hyper- (i.e., group-level) parameters. For each estimation, we obtained 8,000 posterior samples per parameter (= 2,000 iterations × 4 chains) and checked the convergence of the MCMC simulations ($\hat{R}$s < 1.1; Monte Carlo *SE*s < 0.1; effective sample sizes > 0.1). Stan[7] 2.28.0 in R[8] 4.1.0 was used for the analysis.

Cognitive models

Risky decision making (mean-variance utility function)

To model participants' choices in the gambling task, we used the power utility function (Eq. 1) and mean-variance utility function and then compared the performances of the models (see "Model comparisons using LOOIC and WAIC" and Supplementary Table S2 below). In the mean-variance utility function, the utility of option x, $u(x)$, follows

$$u(x) = E(x) + \rho V(x), \tag{S1}$$

where $E(x)$ (= rp_{reward}) is the expected value, and $V(x)$ [= $r^2 p_{\text{reward}}(1 - p_{\text{reward}})$] is the risk of the option; r is the reward magnitude, and p_{reward} is the reward probability (Eq. 1). The participant's risk preference, ρ, is negative if risk averse and positive if risk seeking.

Confidence calibration

As mentioned in the Methods, we modeled participants' confidence calibration and compared

the performance of six probability weighting functions [hereafter $w(p)$], which have been widely used in experimental, behavioral, and neuro economics[9–12]. For the model comparison, see "Model comparisons using LOOIC and WAIC" and Supplementary Table S3 below.

We first fitted the null model, in which the objective and subjective accuracy were perfectly calibrated:

$$w(p) = p. \tag{S2}$$

The second candidate was ref.[13]'s 1-parameter function:

$$w(p) = \exp\left(-(-\ln(p))^{\alpha}\right). \tag{S3}$$

The third candidate was ref.[13]'s two-parameter function:

$$w(p) = \exp(-\beta(-\ln(p))^{\alpha}). \tag{S4}$$

The fourth candidate was ref.[14]'s function:

$$w(p) = \frac{p^{\alpha}}{(p^{\alpha} + (1-p)^{\alpha})^{\frac{1}{\alpha}}}. \tag{S5}$$

The fifth candidate was ref.[9]'s function:

$$w(p) = \frac{p^{\alpha}}{(p^{\alpha} + (1-p)^{\alpha})^{\beta}}. \tag{S6}$$

The sixth candidate was ref.[6]'s function (Eq. 4). This candidate outperformed the other functions in terms of prediction (Supplementary Table S3).

Ordered logistic regression on confidence ratings

To confirm whether participants' subjective accuracy (q in Eq. 4) explains the confidence

ratings in the solo block better than the objective accuracy (p calculated from individual estimates of ε and λ), we further performed an ordered logistic regression, which predicts participant i's probability of rating confidence as k at trial t:

$$P(k)_{i,t} = \begin{cases} 1 - \text{logit}^{-1}(\eta_{i,t} - c_{1,i}), & \text{if } k = 1, \\ \text{logit}^{-1}(\eta_{i,t} - c_{k-1,i}) - \text{logit}^{-1}(\eta_{i,t} - c_{k,i}), & \text{if } 1 < k < 6, \text{and} \\ \text{logit}^{-1}(\eta_{i,t} - c_{5,i}), & \text{if } k = 6, \end{cases} \quad \text{(S7)}$$

where $c_{1\text{–}5}$ are the cut points and η is a predictor given by

$$\eta_{i,t} = \beta_{x,i} Z_{x,i,t}, \quad \text{(S8)}$$

where x is q or p, Z is the normalized x for each participant, and β_x is the coefficient.

Settings of the prior distributions

For each parameter estimation, we set the priors as follows. In the priors, i indicates a participant; $\mu_{(.)}$ and $\sigma_{(.)}$ are the hyper parameters, which control individual differences.

Risky decisions in the gambling task

To stabilize the parameter estimation, we rescaled the magnitude of the reward (JPY) by dividing it by 1,000.

Power utility function (Eqs. 1 and 2)

$$\mu_\rho \sim \text{Cauchy}(0, 5);\ \sigma_\rho \sim \text{StudentT}(4, 0, 1);\ \rho_i \sim \text{Normal}(\mu_\rho, \sigma_\rho);$$

$$\mu_\tau \sim \text{Cauchy}(0, 5);\ \sigma_\tau \sim \text{StudentT}(4, 0, 1);\ \tau_i \sim \text{Normal}(\mu_\tau, \sigma_\tau).$$

Mean-variance utility function (Eq. 2 and Supplementary Eq. S1)

$$\mu_\rho \sim \text{Cauchy}(0, 5);\ \sigma_\rho \sim \text{StudentT}(4, 0, 1);\ \rho_i \sim \text{Normal}(\mu_\rho, \sigma_\rho);$$

$$\mu_\tau \sim \text{Cauchy}(0, 5);\ \sigma_\tau \sim \text{StudentT}(4, 0, 1);\ \tau_i \sim \text{Normal}(\mu_\tau, \sigma_\tau).$$

Stochastic updating model (Eq. 3)

$$\mu_\varepsilon \sim \text{Cauchy}(0,5);\ \sigma_\varepsilon \sim \text{StudentT}(4,0,1);\ \varepsilon_i \sim \text{Normal}(\mu_\varepsilon, \sigma_\varepsilon);$$

$$\mu_\lambda \sim \text{Cauchy}(0,5);\ \sigma_\lambda \sim \text{StudentT}(4,0,1);\ \lambda_i \sim \text{Normal}(\mu_\lambda, \sigma_\lambda).$$

Confidence calibration

To stabilize the parameter estimation, we rescaled the magnitude of the reward (JPY) by dividing it by 1,000.

Null model (Eq. 2 and Supplementary Eq. S2)

$$\mu_\tau \sim \text{Cauchy}(0,5);\ \sigma_\tau \sim \text{StudentT}(4,0,1);\ \lambda_i \sim \text{Normal}(\mu_\tau, \sigma_\tau).$$

Prelec one-parameter function (Eq. 2 and Supplementary Eq. S3)

$$\mu_\alpha \sim \text{Cauchy}(0,5);\ \sigma_\alpha \sim \text{StudentT}(4,0,1);\ \alpha_i \sim \text{Normal}(\mu_\alpha, \sigma_\alpha);$$

$$\mu_\tau \sim \text{Cauchy}(0,5);\ \sigma_\tau \sim \text{StudentT}(4,0,1);\ \lambda_i \sim \text{Normal}(\mu_\tau, \sigma_\tau).$$

Prelec two-parameter function (Eq. 2 and Supplementary Eq. S4)

$$\mu_\alpha \sim \text{Cauchy}(0,5);\ \sigma_\alpha \sim \text{StudentT}(4,0,1);\ \alpha_i \sim \text{Normal}(\mu_\alpha, \sigma_\alpha);$$

$$\mu_\beta \sim \text{Cauchy}(0,5);\ \sigma_\beta \sim \text{StudentT}(4,0,1);\ \beta_i \sim \text{Normal}(\mu_\beta, \sigma_\beta);$$

$$\mu_\tau \sim \text{Cauchy}(0,5);\ \sigma_\tau \sim \text{StudentT}(4,0,1);\ \tau_i \sim \text{Normal}(\mu_\tau, \sigma_\tau).$$

Tversky–Kahneman function (Eq. 2 and Supplementary Eq. S5)

$$\mu_\alpha \sim \text{Cauchy}(0,5);\ \sigma_\alpha \sim \text{StudentT}(4,0,1);\ \alpha_i \sim \text{LogNormal}(\mu_\alpha, \sigma_\alpha);$$

$$\mu_\tau \sim \text{Cauchy}(0,5);\ \sigma_\tau \sim \text{StudentT}(4,0,1);\ \tau_i \sim \text{Normal}(\mu_\tau, \sigma_\tau).$$

Wu–Gonzalez function (Eq. 2 and Supplementary Eq. S6)

$$\mu_\alpha \sim \text{Cauchy}(0,1);\ \sigma_\alpha \sim \text{StudentT}(10,0,1);\ \alpha_i \sim \text{Normal}(\mu_\alpha, \sigma_\alpha);$$

$$\mu_\beta \sim \text{Cauchy}(0,1);\ \sigma_\beta \sim \text{StudentT}(10,0,1);\ \beta_i \sim \text{Normal}(\mu_\beta, \sigma_\beta);$$

$$\mu_\tau \sim \text{Cauchy}(0,5);\ \sigma_\tau \sim \text{StudentT}(4,0,1);\ \tau_i \sim \text{Normal}(\mu_\tau, \sigma_\tau).$$

Goldstein–Einhorn function (Eqs. 2 and 4)

$$\mu_\alpha \sim \text{Cauchy}(0,1);\ \sigma_\alpha \sim \text{StudentT}(10,0,1);\ \alpha_i \sim \text{Normal}(\mu_\alpha, \sigma_\alpha);$$

$$\mu_\beta \sim \text{Cauchy}(0,1);\ \sigma_\beta \sim \text{StudentT}(10,0,1);\ \beta_i \sim \text{Normal}(\mu_\beta, \sigma_\beta);$$

$$\mu_\tau \sim \text{Cauchy}(0,5);\ \sigma_\tau \sim \text{StudentT}(4,0,1);\ \tau_i \sim \text{Normal}(\mu_\tau, \sigma_\tau).$$

Statistical analyses

Logistic regression (Eq. 6)

$$\mu_{\beta_1} \sim \text{Cauchy}(0,5);\ \sigma_{\beta_1} \sim \text{StudentT}(4,0,1);\ \beta_{1,i} \sim \text{Normal}(\mu_{\beta_1}, \sigma_{\beta_1});$$

$$\mu_{\beta_r} \sim \text{Cauchy}(0,5);\ \sigma_{\beta_r} \sim \text{StudentT}(4,0,1);\ \beta_{r,i} \sim \text{Normal}(\mu_{\beta_r}, \sigma_{\beta_r});$$

$$\mu_{\beta_v} \sim \text{Cauchy}(0,5);\ \sigma_{\beta_v} \sim \text{StudentT}(4,0,1);\ \beta_{v,i} \sim \text{Normal}(\mu_{\beta_v}, \sigma_{\beta_v});$$

$$\beta_\rho \sim \text{Cauchy}(0,5);\ \beta_\gamma \sim \text{Cauchy}(0,5);\ \beta_c \sim \text{Cauchy}(0,5);\ \beta_\lambda \sim \text{Cauchy}(0,5).$$

Ordered logistic regression (Supplementary Eqs. S7 and S8)

$$\mu_{c_{1-5}} \sim \text{Cauchy}(0,5);\ \sigma_{c_{1-5}} \sim \text{StudentT}(4,0,1);\ c_{1-5,i} \sim \text{Normal}(\mu_{c_{1-5}}, \sigma_{c_{1-5}});$$

$$\mu_{\beta_x} \sim \text{Cauchy}(0,5);\ \sigma_{\beta_x} \sim \text{StudentT}(4,0,1);\ \beta_{x,i} \sim \text{Normal}(\mu_{\beta_x}, \sigma_{\beta_x}).$$

Model comparisons using LOOIC and WAIC

To compare the prediction performances of the models, we computed (i) the information criterion by approximate leave-one-out cross-validation (LOOIC)[15] and (ii) the widely applicable information criterion (WAIC)[16]. In both indices, a lower value indicates a better model performance in terms of prediction. The "loo" package[17] 2.4.1 in R[8] was used to

calculate these indices.

Supplementary Table S2 shows that for the risky choices in the gambling task, the power utility function (Eq. 1) was better than the mean-variance utility function (Supplementary Eq. S1). Supplementary Table S3 shows that participants' confidence calibration was predicted best by ref.[6]'s function (Eq. 4). Supplementary Table S4 shows that the subjective accuracy (q in Eq. 4) predicts participants' confidence ratings better than the objective accuracy (p estimated from individual estimates of ε and λ). The statistical results remained unchanged between the LOOIC and the WAIC.

Posterior predictive checking

To check the fit of the best models selected above to participants' behavior, we performed posterior predictive checking[18–20] by generating posterior predictive samples and comparing them to the actual participants' behavior in the experiment (Supplementary Figs. S7 and S8). As shown in the figures, the posterior predictive samples do not deviate significantly from the actual data, thus providing support for the validity of our models.

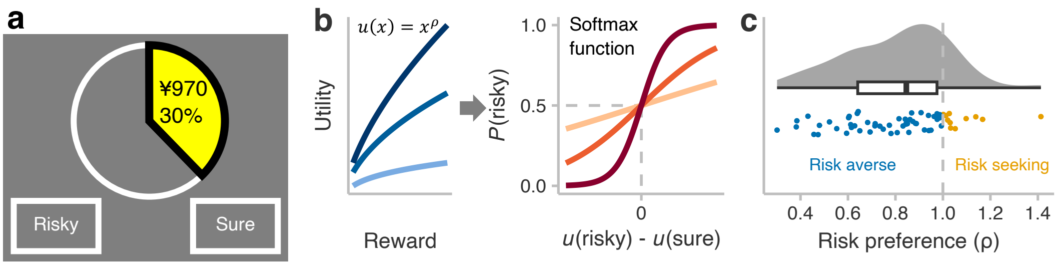

Figure S1. Gambling task and the model for risky decisions. **(a) An example of stimuli in the gambling task.** The pie chart indicates the reward magnitude and probability of the risky option. The yellow area shows the reward probability, which is 30% in this example. The left and right positions of the options were randomized across trials. Participants chose the left (right) option with the Q (P) key on a keyboard. **(b) Model for risky decisions in the gambling task and the solo block of the orientation-judgment task.** The utility function is assumed to be a power function (Eq. 1), and the probability of choosing the risky option is assumed to follow a softmax (logistic) function (Eq. 2). **(c) Distribution of participants' risk preferences.** Fifty-two participants (i.e., 83% of participants) were risk averse. Each dot indicates one participant's data.

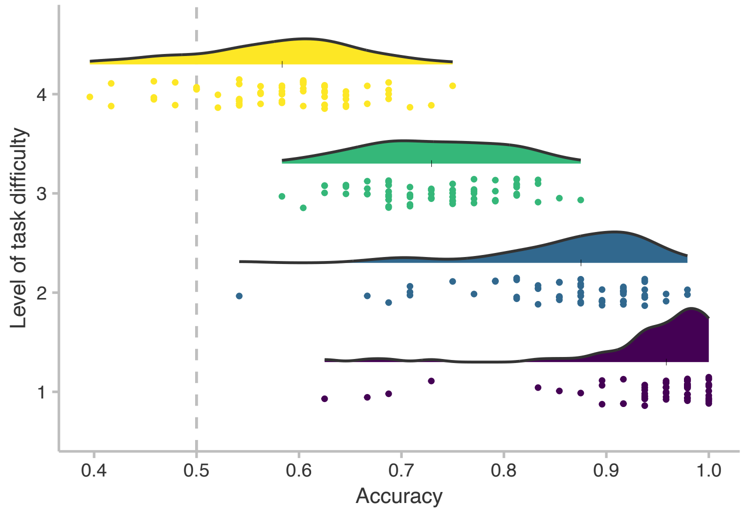

Figure S2. Distribution of individual accuracy as a function of the level of task difficulty. For Levels 1–3, no participants' accuracy was less than chance level, 0.5; however, for Level 4, there were nine participants whose accuracy was less than 0.5, and three participants whose accuracy was exactly 0.5. Each dot indicates one participant's data, and the accuracy is collapsed across the solo and opt-in/out blocks.

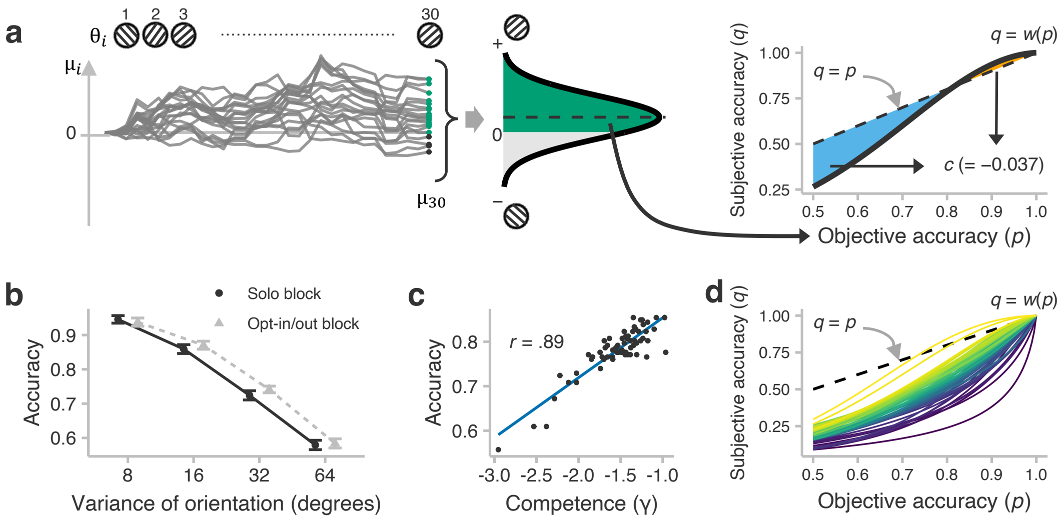

Figure S3. Estimating competence and confidence. **(a) Illustrations of the stochastic updating model and confidence calibration.** Participants updated the noisy estimate of the orientation (Eq. 3). Since the updating process involves random noise, it takes different paths probabilistically (i.e., the gray lines in the left panel). Because the random noise from the normal distribution is repeatedly added in this updating process, the final outputs (μ_{30}: the green or black dots) also follow the normal distribution (the center panel). We assumed that participants could compute the probability of being correct (i.e., the objective accuracy, *p*) according to the cumulative probability of the distribution (the green-filled area; see ref.[2] for details). Participants were also assumed to perceive the objective accuracy with biases, which were modeled using the probability weighting function, $w(p)$ (Eq. 4). The index of each participant's confidence was computed by integrating the area surrounded by $w(p)$ and the 45-degree line ($q = p$, which indicates perfect calibration between the objective and subjective accuracy; the right panel). The blue (orange) area shows whether the participant perceived their own objective accuracy as lower (higher) than it actually was. The sum of these areas, *c*, was used as the index of the participant's overall confidence. In this example, $c = -0.037$ indicates that the participant was a little underconfident. **(b) Accuracy as a function of block and variance of orientations (*M* ± *SEM* across participants).** No significant difference was

observed between the blocks, whereas the accuracy decreased as the variance increased. **(c) Positive correlation between γ and accuracy.** Clearly, γ is a good indicator of participants' competence. The accuracy is collapsed across blocks and levels of task difficulty. **(d) Participants' confidence calibration.** Each curve indicates each participant's confidence calibration curve. A line color closer to yellow indicates that the participant's c is larger.

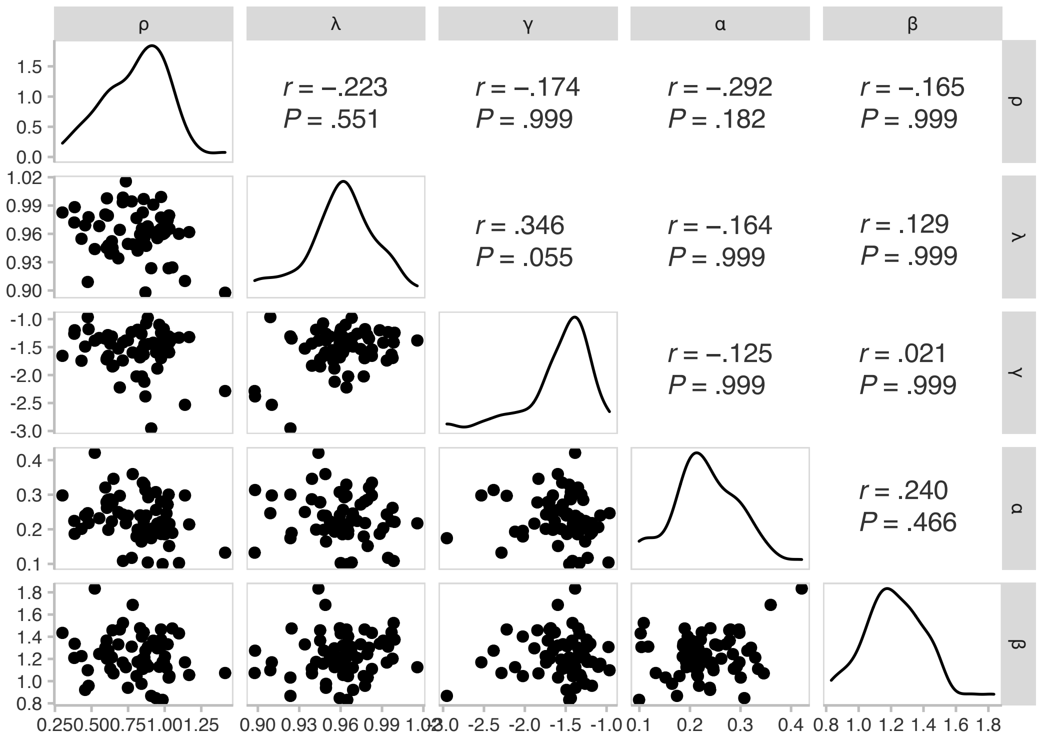

Figure S4. Correlations among the estimates of cognitive parameters. The diagonal panels show the distributions of the estimates, and the upper panels show the Pearson correlations. *P* values are adjusted by the Holm–Bonferroni method.

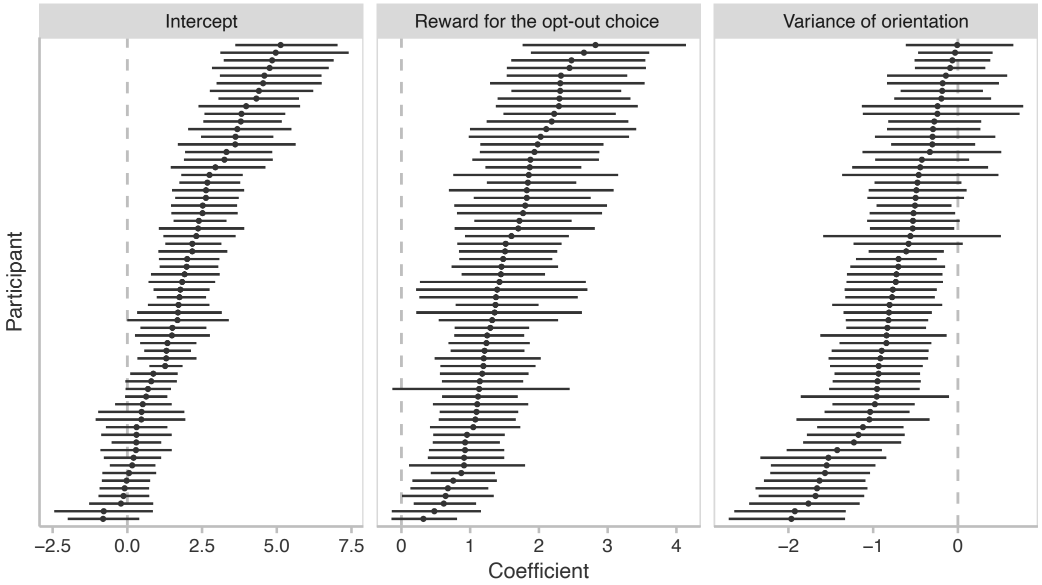

Figure S5. Individual coefficients for each variable in the logistic regression on the opt-in/out choices. The points indicate the median of the posterior samples, and the error bars indicate the 95% credible intervals.

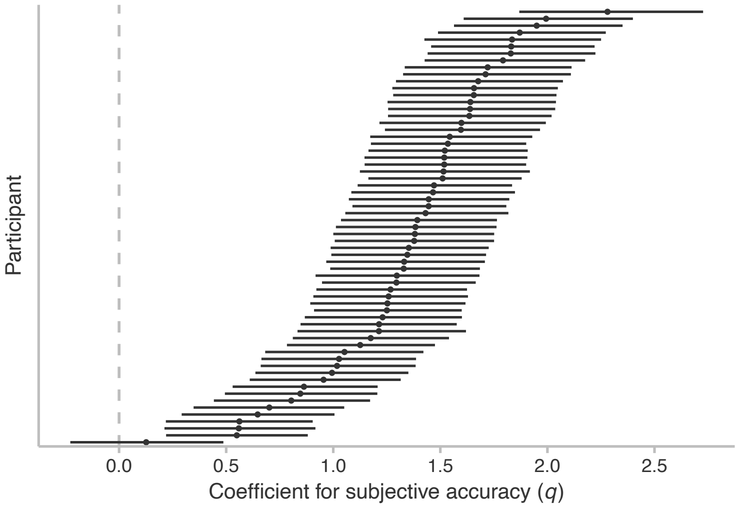

Figure S6. Individual coefficients for the subjective accuracy in the ordered logistic regression on confidence ratings. The points indicate the medians of the posterior samples, and the error bars indicate the 95% credible intervals. For 62 of 63 participants, the subjective accuracy had a positive effect on their confidence ratings.

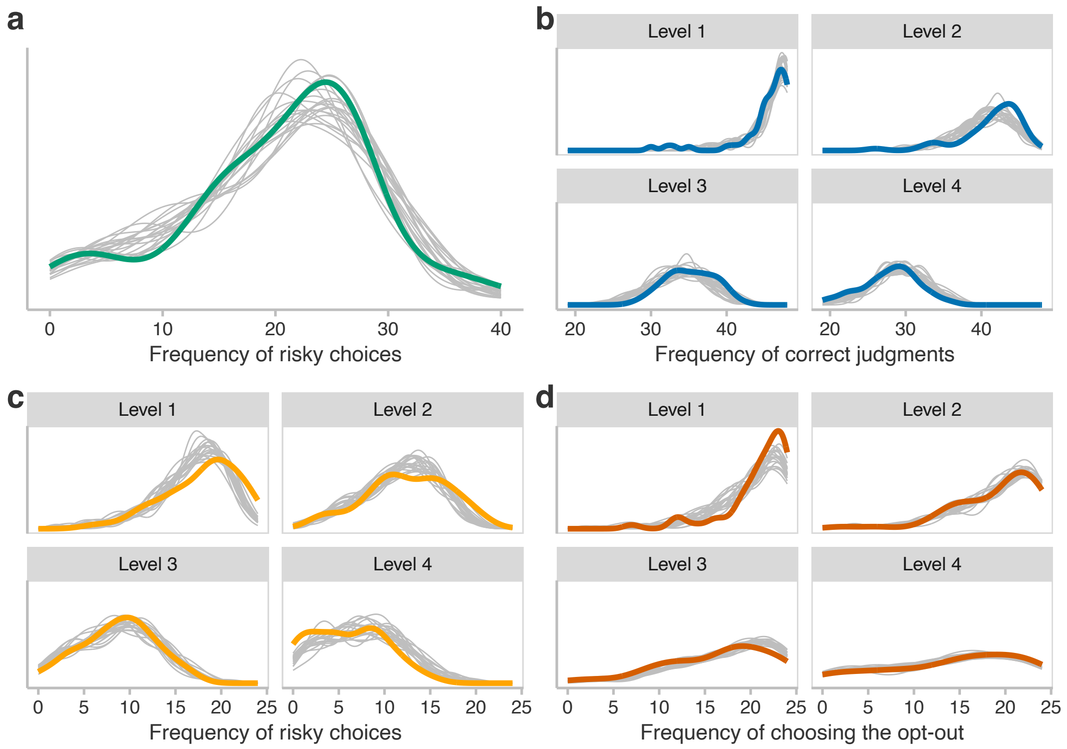

Figure S7. Posterior predictive checking. In each density plot, a bold line indicates the actual data from the experiment, and the 20 gray lines indicate samples generated from the posterior distributions. The "level" on the top of each panel indicates the level of task difficulty. **(a) Frequency of risky choices in the gambling task.** The samples are generated from the power utility function and softmax function (Eqs. 1 and 2; Supplementary Fig. S1b). **(b) Frequency of correct judgments in the orientation-judgment task.** The samples are generated from the stochastic updating model (Eq. 3; the left panel in Supplementary Fig. S3a). **(c) Frequency of risky choices in the solo block.** The samples are generated from the probability weighting function (Eq. 4) and the models on risky choices (Eqs. 1 and 2). **(d) Frequency of the opt-out choice in the opt-in/out block.** The samples are generated from the logistic regression (Eq. 6).

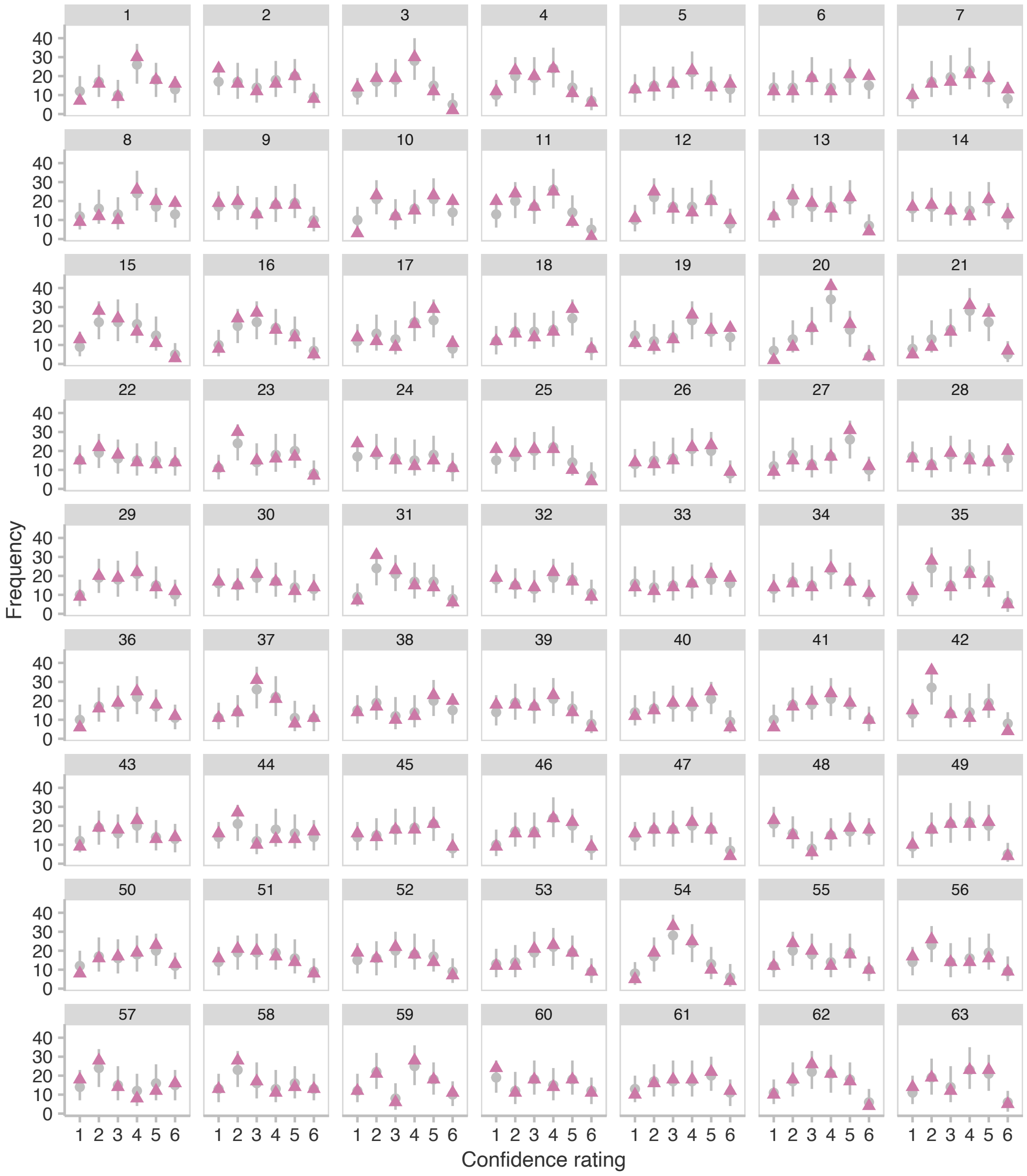

Figure S8. Posterior predictive checking on the ordered logistic regression predicting participants' confidence ratings from the subjective accuracy. Each panel shows one participant's data. The pink triangles indicate the actual frequency of rating confidence as 1–6, the gray circles indicate the medians of the posterior predictive samples, and the gray lines indicate the 95% credible intervals.

Table S1. Forty-seven risky options in the gambling task

Reward probability	Reward (JPY)	Reward probability	Reward (JPY)	Reward probability	Reward (JPY)	Reward probability	Reward (JPY)
.3	970	.4	830	.5	730	1.0	300
.3	1,040	.4	870	.5	760	1.0	700
.3	1,120	.4	920	.5	800		
.3	1,220	.4	990	.5	840		
.3	1,340	.4	1,060	.5	880		
.3	1,490	.4	1,150	.5	940		
.3	1,670	.4	1,250	.5	1,000		
.3	1,890	.4	1,380	.5	1,080		
.3	2,180	.4	1,530	.5	1,170		
.3	2,540	.4	1,720	.5	1,270		
.3	3,010	.4	1,960	.5	1,410		
.3	3,640	.4	2,260	.5	1,570		
.3	4,480	.4	2,650	.5	1,770		
.3	5,650	.4	3,160	.5	2,020		
.3	7,290	.4	3,840	.5	2,340		

Note. Trial order was randomized across participants. As in ref.[21], we did not use probabilities less than .3 to reduce the confounding effects of a distorted subjective probability. The two catch trials, in which the winning probability was 1.0, were excluded from the analysis.

Table S2. LOOIC and WAIC for the models on participants' risky choices in the gambling task

Model	LOOIC	WAIC
Power utility (Eq. 1)	1,945.0	1,937.1
Mean-variance (Eq. S1)	2,305.7	2,275.3

Note. LOOIC = The information criterion by approximate leave-one-out cross-validation; WAIC = the widely applicable information criterion.

Table S3. LOOIC and WAIC for the models on participants' confidence calibration

Model	LOOIC	WAIC
Goldstein–Einhorn (Eq. 4)	5,376.4	5,375.8
Prelec two-parameter (Eq. S4)	5,394.2	5,393.6
Tversky–Kahneman (Eq. S5)	5,428.9	5,428.4
Wu–Gonzalez (Eq. S6)	5,448.6	5,448.2
Prelec one-parameter (Eq. S3)	5,874.3	5,873.8
Null (Eq. S2)	7,373.0	7,372.9

Note. LOOIC = The information criterion by approximate leave-one-out cross-validation; WAIC = the widely applicable information criterion.

Table S4. LOOIC and WAIC for the models on participants' confidence ratings in the solo block

Model	LOOIC	WAIC
q (subjective accuracy)	18,553.6	18,552.9
p (objective accuracy)	18,662.3	18,661.7

Note. LOOIC = The information criterion by approximate leave-one-out cross-validation; WAIC = the widely applicable information criterion.

Supplementary References

1. Pedroni, A. *et al.* The risk elicitation puzzle. *Nat. Hum. Behav.* **1**, 803–809 (2017).
2. Navajas, J. *et al.* The idiosyncratic nature of confidence. *Nat. Hum. Behav.* **1**, 810–818 (2017).
3. Blavatskyy, P. R. Betting on own knowledge: experimental test of overconfidence. *J. Risk Uncertain.* **38**, 39–49 (2009).
4. Clark, J. & Friesen, L. Overconfidence in forecasts of own performance: an experimental study. *Econ. J.* **119**, 229–251 (2009).
5. Murad, Z., Sefton, M. & Starmer, C. How do risk attitudes affect measured confidence? *J. Risk Uncertain.* **52**, 21–46 (2016).
6. Goldstein, W. M. & Einhorn, H. J. Expression theory and the preference reversal phenomena. *Psychol. Rev.* **94**, 236–254 (1987).
7. Carpenter, B. *et al.* Stan: a probabilistic programming language. *J. Stat. Softw.* **76**, 1–32 (2017).
8. R Core Team. *R: a language and environment for statistical computing*. https://www.R-project.org/ (2022).
9. Wu, G. & Gonzalez, R. Curvature of the probability weighting function. *Manage. Sci.* **42**, 1676–1690 (1996).
10. Gonzalez, R. & Wu, G. On the shape of the probability weighting function. *Cogn. Psychol.* **38**, 129–166 (1999).
11. Stott, H. P. Cumulative prospect theory's functional menagerie. *J. Risk Uncertain.* **32**, 101–130 (2006).
12. Hsu, M., Krajbich, I., Zhao, C. & Camerer, C. F. Neural response to reward anticipation under risk is nonlinear in probabilities. *J. Neurosci.* **29**, 2231–2237 (2009).
13. Prelec, D. The probability weighting function. *Econometrica* **66**, 497–527 (1998).

14. Tversky, A. & Kahneman, D. Advances in prospect theory: cumulative representation of uncertainty. *J. Risk Uncertain.* **5**, 297–323 (1992).

15. Vehtari, A., Gelman, A. & Gabry, J. Practical Bayesian model evaluation using leave-one-out cross-validation and WAIC. *Stat. Comput.* **27**, 1413–1432 (2017).

16. Watanabe, S. & Opper, M. Asymptotic equivalence of Bayes cross validation and widely applicable information criterion in singular learning theory. *J. Mach. Learn. Res.* **11**, 3571–3594 (2010).

17. Vehtari, A. *et al. loo: Efficient leave-one-out cross-validation and WAIC for Bayesian models*. https://mc-stan.org/loo/ (2020).

18. Box, G. E. P. Sampling and Bayes' inference in scientific modelling and robustness. *J. R. Stat. Soc. Ser. A* **143**, 383–430 (1980).

19. Rubin, D. B. Bayesianly justifiable and relevant frequency calculations for the applied statistician. *Ann. Stat.* **12**, 1151–1172 (1984).

20. Gelman, A., Meng, X.-L. & Stern, H. Posterior predictive assessment of model fitness via realized discrepancies. *Stat. Sin.* **6**, 733–760 (1996).

21. Suzuki, S., Jensen, E. L. S., Bossaerts, P. & O'Doherty, J. P. Behavioral contagion during learning about another agent's risk-preferences acts on the neural representation of decision-risk. *Proc. Natl. Acad. Sci. U. S. A.* **113**, 3755–3760 (2016).
